# Outcomes of an Online Virtual Boot Camp to Prepare Fourth-Year Medical Students for a Successful Transition to Internship

**DOI:** 10.7759/cureus.8558

**Published:** 2020-06-11

**Authors:** Lea M Monday, Anthony Gaynier, Madeline Berschback, David Gelovani, Henry Y Kwon, Sahrish Ilyas, Asra N Shaik, Diane L Levine

**Affiliations:** 1 Internal Medicine, Detroit Medical Center, Detroit, USA; 2 Internal Medicine, John D. Dingell VA Medical Center, Detroit, USA; 3 Medical Education, Wayne State University School of Medicine, Detroit, USA; 4 Internal Medicine, Wayne State University School of Medicine, Detroit, USA; 5 Internal Medicine, Wayne State University, Detroit Medical Center, Detroit, USA

**Keywords:** undergraduate medical education, medical students, transition, boot camp, internship, residency preparation, remote learning, online teaching, non-surgical, capstone course

## Abstract

Introduction

Changes in medical education and health care delivery have limited the ability of fourth-year medical students to perform the role of an intern prior to graduating from medical school. To address this issue, many schools have instituted residency preparation courses (sometimes referred to as boot camps) particularly for students entering surgical fields. Courses for students entering nonprocedural fields are less common and most assess increases in self-reported confidence without providing objective evidence of a gain in knowledge or skills improvement.

Materials and Methods

We used a Plan, Do, Study, Act (PDSA) model to develop and pilot cycle 1 of a nonprocedural internship preparation elective in 2019. Feedback was used to refine the course and map sessions to core competencies outlined by the Accreditation Council of Graduate Medical Education (ACGME) for PDSA cycle 2. The curriculum was adapted for remote synchronous delivery due to the coronavirus pandemic in spring 2020 using a combination of didactic lectures containing embedded polls and case-based role play responses using a chat box. Students completed anonymous surveys assessing self-perceived levels of confidence, as well as an objective comprehensive assessment after course completion.

Results

A total of 89 students participated in the course. Pre-session confidence was lowest for transfusion medicine, handling pages from nursing while on call, and knowledge of the role of a chief resident. A statistically significant increase in median scores for self-reported knowledge or confidence was seen in all sessions. The percentage of students reporting that they were either confident or extremely confident also increased significantly after each session (p<0.001 for all). All sessions analyzed were rated as useful or extremely useful by more than half of the students, and 94% of the students scored 70% or higher on the comprehensive course assessment.

Conclusions

An online virtual synchronous boot camp increased students’ confidence in handling common topics encountered during residency and demonstrated an appropriate gain in knowledge using a comprehensive assessment. We were able to adapt our curriculum to a remote model and will likely keep several sessions in an online format in the future.

## Introduction

The goal of medical schools is to prepare students to be doctors ready for postgraduate training in their field of choice. Since the publication of Flexner’s 2 x 2 model in 1910, undergraduate medical education (UME) curriculum has been divided into two phases: the preclinical phase, which is focused on pathophysiology and medical theory, and the clinical phase, which is focused on training in surgical and non-surgical fields. In the United States, the fourth year of medical school varies in content and structure to maximize student freedom to choose elective courses and non-clinical pursuits [1-3]. Ideally, students will have opportunities during clerkship to care for patients from admission until discharge and immersed in patient care as if they are already interns. However, the degree of student immersion and experience deviates from this ideal due to several factors. Patient length of stay has shortened, procedures are increasingly outsourced to interventional subspecialists, patient handoffs are frequent, and care is generally more fragmented than decades ago [4]. In addition, changes in medical education such as duty hour regulation and limited privileges within the electronic medical record (EMR) reduce student autonomy. The degree of student experience with the EMR varies. In one recent survey, fewer than 10% of students on their obstetrics clerkship had entered orders and less than half (47%) had written a history and physical note in the patient chart [5]. In another large survey of around 16,600 graduating medical students, 84% had entered information of some type into the EMR on their internal medicine clerkship or subinternship; however, 43% had never entered admission orders [6]. Yet students are expected to step into residency on July 1 and care for patients, including night shifts and with varying degrees of supervision and support. These challenges result in increased stress for interns, educational difficulties for program directors, and, most importantly, potentially unsafe care for patients.

In order to address the difficulties in the transition to internship, some medical schools have created capstone courses focused on communication, common problems, procedural skills, or some combination of topics for students entering internship. Elective courses for students entering dentistry [7], surgery [8-12], and obstetrics and gynecology [13-15] have been shown to increase confidence in procedural skills and communication. Similar courses in non-surgical specialties are not as common; however, their numbers are increasing. Boot camp experiences have been replicated in family medicine [16], pediatrics [16,17], emergency medicine [18], and with students entering a mix of fields including internal medicine [19-21]. A common result is an increase in the level of confidence or preparedness reported among students. In a 2014 meta-analysis, boot camps were shown to be an effective educational strategy to improve learners' clinical skills, knowledge, and confidence [22].

Medical students graduating in 2020 were removed from all direct patient care and in-person educational activities due to the coronavirus disease 2019 (COVID-19) pandemic. Unfortunately, this drastically altered the content and delivery of courses at medical schools throughout the United States, including internship preparation courses. Remote delivery of one boot camp course has been briefly reported for students entering pediatric residency [23]. As of May 2020, there is no published work describing the logistics and results of executing a non-surgical internship preparation course open to all medical students regardless of match specialty.

We describe a one-month intensive course entitled, “Internship Boot Camp”, which was designed after a previous pilot and adapted to be delivered remotely during the COVID-19 pandemic. This online virtual synchronous interactive elective was taught by faculty, residents, and near peers, with the goal of assisting fourth-year medical students with the transition to internship. The goals of this study were to determine the effectiveness of the Internship Boot Camp on three measures: (1) level of confidence about entering their internship, (2) ability to develop a framework for responding to common challenges, and (3) ability to demonstrate an appropriate gain in clinical knowledge using a comprehensive post-course assessment. Here, we report the outcomes of a non-surgical intern preparatory course.

## Materials and methods

Pilot development and PDSA cycle 1: first nonprocedural boot camp

**Figure 1:**
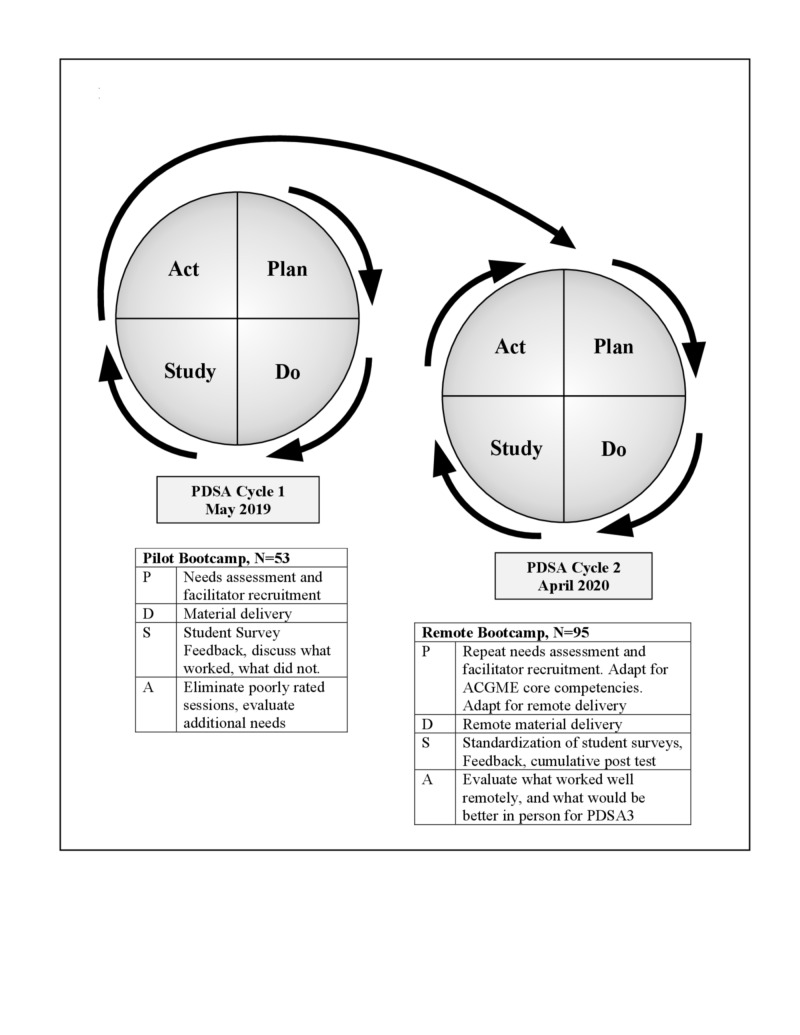
Intern Boot Camp Design Using a Rolling PDSA Cycle ACGME, Accreditation Council for Graduate Medical Education; PDSA, Plan, Do, Study, Act

Faculty from the Department of Surgery at our institution had been offering a procedural boot camp focused on laparoscopic skills and knot tying since 2005; however, no such course existed for students entering nonprocedural fields. Starting in spring 2019, we developed and piloted a nonprocedural preparation elective utilizing PDSA (Plan, Do, Study, Act) methodology (Figure 1).

Fifty-three students from the Wayne State University School of Medicine who matched into a variety of non-surgical residencies were offered an ungraded elective entitled, “Intern Boot Camp.” Before developing the boot camp curriculum, a needs assessment (a systematic process for determining and addressing needs or "gaps" existing in the knowledge and skills of medical students) was conducted through a voluntary online survey. Input was provided by clerkship directors, residency program directors, residents, recent graduates, and our graduating medical students. Next, a targeted needs assessment was performed to determine specific deficiencies, and course sessions were created with specific goals and objectives to meet the deficiencies identified. For example, if program directors answered that new interns did not know how to write admission orders, the specific deficiencies were asked on the targeted needs assessment (such as not understanding order sets, or diet and activity orders). This course was approved by the Curriculum Management Committee and added to the course catalog for April and May of 2019. Faculty, chief medical residents, and residents taught each session. Oversight was provided by the course director. Course content was delivered using a variety of modalities including a combination of didactic PowerPoint lectures, procedures on lightly preserved cadavers, a session on how to teach and give effective feedback, and case-based role play for handling difficult conversations, medical emergencies, and simulated pages from nursing. Lastly, students completed 12 symptom-based modules (which covered common clinical scenarios encountered on call) through the WISE-On-Call program distributed by Aquifer® on behalf of New York University (NYU) School of Medicine [24]. After the pilot, a post-course survey was conducted to evaluate students’ self-assessed learning achieved through the course and to learn where improvement was needed. These data were taken into consideration while adjusting and creating the schedule for April 2020. Institutional Review Board approval was not obtained as students participated voluntarily, topics covered constituted routine education, and the assessment of the learners was part of the normal course evaluation process.

PDSA cycle 2 and adaptation for remote delivery

In early 2020, planning began to scale this activity up to approximately one half of the class for April 1, 2020 (Figure 1), in preparation for delivery to all 300 students by spring 2021. Student feedback was used to adjust session topics, and the curriculum was redeveloped so that content was divided into six interrelated domains based on the Accreditation Council for Graduate Medical Education (ACGME) core competencies: patient care, medical knowledge, practice-based learning and improvement, interpersonal communication skills, professionalism, and systems-based practice (Table 1) [25].

**Table 1:** ACGME Six Core Competencies Source: Outcome Project©, ACGME 2007. https://www.ecfmg.org/echo/acgme-core-competencies.html ACGME, Accreditation Council for Graduate Medical Education

Core Competency	Description
Patient care	Residents must be able to provide patient care that is compassionate, appropriate, and effective for the treatment of health problems and the promotion of health.
Medical knowledge	Residents must be able to demonstrate knowledge about established and evolving biomedical, clinical, and cognate (e.g. epidemiological and social-behavioral) sciences, and the application of this knowledge to patient care.
Practice-based learning and improvement	Residents must be able to investigate and evaluate their patient care practices, appraise and assimilate scientific evidence, and improve their patient care practices.
Interpersonal communication skills	Residents must be able to demonstrate interpersonal and communication skills that result in effective information exchange and teaming with patients, patients’ families, and professional associates.
Professionalism	Residents must be able to demonstrate a commitment to carrying out professional responsibilities, adherence to ethical principles, and sensitivity to a diverse patient population.
Systems-based practice	Residents must be able to demonstrate an awareness of and responsiveness to the larger context and system of health care and the ability to effectively call on system resources to provide care that is of optimal value.

The curriculum was also designed to reinforce the American Academy of Medical Colleges (AAMC) 13 Core Entrustable Professional Activity’s (EPAS) whenever possible [26]. Content was delivered in 26 sessions (22 mandatory and 4 optional). Detailed mapping of each boot camp session topic with a description and the corresponding ACGME core competency is provided in Table 2.

With the onset of the COVID-19 pandemic, all student face-to-face activities were furloughed on March 17, 2020. The course was rapidly transformed in 14 days to an entirely online curriculum. We adapted content for synchronous remote delivery using the Canvas online learning management system (Instructure®, Salt Lake City, UT, USA). Synchronous sessions used a combination of didactic PowerPoint presentations that were made interactive by adding facilitator polling, multiple-choice questions, and small group or individual writing exercises that were shared with the group through incorporation into the presentation or chat board. For example, students were given a mock patient sign out electronically and had to use this to respond to virtual pages from a nurse for a simulated night-on-call. Students were asked to write a note about some of the overnight events, which were later de-identified and shared with the group for feedback during the “Sign Out” lecture. Procedural training on cadavers was adapted to include an independent review of online procedure videos by students. Training in pronouncing death on a recently deceased cadaver and calling his/her family was adapted to include an interactive session on pronouncing patient death and a reflective reading assignment. An “Ask the Interns” panel discussion was adapted by requesting students to e-mail questions for review by three high functioning interns who then took turns answering them through video chat and the live message board. Pertinent hot topics were added, including a session on telehealth and a COVID-19 patient case discussion that included a rich discussion of end-of-life care and the ethics surrounding decision-making with limited resources. In addition to the synchronous remote sessions, students were required to submit written exercises with a focus on compassionate care, wellness, and self-reflection. Students wrote a letter to members of the incoming class of medical students. Additional independent study requirements included 12 Aquifer cases and online naloxone training. Sessions were grouped so that students had a Monday or Friday free each week to facilitate their end-of-year personal tasks such as finalizing housing plans or pre-employment requirements for their upcoming residencies, which may be in different cities or states. Four additional optional sessions were held, one about preparing a professional PowerPoint and three focused on topics pertinent to the care of pediatric patients.

Outcome evaluation methods

For 14 out of 26 sessions, the facilitators created a voluntary pre-session survey through a secure online data collection tool. Medical students were asked to rate their confidence and understanding of requisite knowledge points on the topic before these sessions. Items were graded on a 5-point Likert scale (1 meaning confidence or knowledge was very poor, 3 meaning neutral, and 5 meaning very high). After delivery of each session, students were given a post-survey with one additional question about whether they found the activity useful. A competency-based exam was administered at the conclusion of the course to assess the knowledge gained. Two to three multiple-choice questions were written for each lecture session assessing student competency of objectives identified by the educator. The questions were each mapped to ACGME core competencies and written with varying degrees of difficulty addressing knowledge, application, and problem-solving [25,27]. The resulting 53-question comprehensive assessment was completed by all students except for one who contracted COVID-19. After completing the comprehensive assessment, students were given a link to the correct responses. Pre-/post-survey median scores were analyzed using the Wilcoxon rank-sum test (Mann-Whitney U test) for unpaired non-parametric data. To categorically compare the percentages of students who answered positively about each session, responses in the Likert scale were combined into a 3 x 2 contingency table (positive, neutral, or negative responses), and data were analyzed using the chi-square test. For example, the Likert scale response categories of extremely confident and confident were combined into one, “positive” category. Usefulness of sessions and post-test grades were analyzed with descriptive statistics. All tests were two-tailed, with p < 0.05 as the significance level. Statistical analysis was performed using IBM SPSS Version 25 (IBM Corp., Armonk, NY, USA).

## Results

A total of 89 students and 15 instructors participated remotely. Student learners had matched into a mix of non-surgical residency programs, with internal medicine, emergency medicine, family medicine, being most common. Instructors included faculty, chief residents, and current interns. Pre-surveys were completed by 72-83 students (76-87%), and post-surveys were submitted by 57-78 students (60-82%). Pre-test confidence scores were lowest for the sessions on transfusion medicine, the nursing pager on call activity, and chief medical resident expectations, with less than 10% of students feeling confident or extremely confident in those topics. Pre-session confidence scores were highest in teaching medical students and providing feedback (55% and 69% of students felt at least confident and comfortable, respectively). The percentages of students who answered that they were confident or extremely confident in the subject matter before and after each session are summarized in Table 3. All synchronous sessions resulted in a measurable increase in the portion of students reporting confidence or extreme confidence in the subject matter. The largest increases in confidence (over 75% increase) were seen in the session about when go to your program director, chief medical resident expectations, and what do after a patient dies, followed by the session on using blood products (66% increase).

**Table 2:** Percentage of Self-Reported Confidence or Knowledge Scores in the High or Extremely High Category Before and After Boot Camp Virtual Sessions All values are rounded to the nearest 1%. To compare the percentages of students who answered positively for each question, the response categories in the Likert scale were combined into a 3 x 2 contingency table (positive, neutral, and negative), and data were analyzed using the chi-square test.

Session Title	Confidence or Knowledge Statement	Pre %	Post %	Delta	p-Value
Your Program Director	I can name several specific instances when I should go directly to the program director with an issue/concern.	14	93	79	<0.001
Chief Resident Expectations	I am knowledgeable of the role of a chief resident.	8	85	77	<0.001
After a Patient Dies	I know how to complete a death certificate	11	88	77	<0.001
Blood Products/Transfusion	I am confident that I know when to order certain blood products such as leukocyte reduced versus irradiated.	1	67	66	<0.001
Concussion/Head Trauma	I am comfortable with the protocol for on-field head or spinal trauma.	22	81	59	<0.001
Common Clinical Scenarios	I am confident in managing common clinical scenarios.	19	77	58	<0.001
BEEP! A Night-on-Call: Nursing Pager Activity	I am confident in handling pages from a nurse while I am on call.	4	59	55	<0.001
Sign Out/Transitions of Care	I am confident in signing out my patients to my peers.	24	77	53	<0.001
Admission Process and Writing Orders	I am confident in writing admission orders independently.	17	69	52	<0.001
Clinical Reasoning	I am confident in my ability to find and apply an appropriate illness script.	27	79	52	<0.001
Clinic/Ambulatory Tips	I am confident in caring for patients efficiently in the clinic setting.	27	74	47	<0.001
Clinical Documentation	I am confident in my clinical documentation.	46	89	43	<0.001
Teaching Students	I am confident in my teaching abilities.	55	92	37	<0.001
Telehealth	I am comfortable participating in telehealth as a health care provider.	39	75	36	<0.001

Median confidence scores before and after the sessions are presented in Table 4. Before the session, median scores were lowest regarding knowledge about the role of a chief resident, confidence writing admission orders independently, using blood products, and how to fill out a death certificate. Baseline median confidence scores were highest for teaching medical students and knowledge about how to give students feedback. A measurable increase in numerical confidence or knowledge scores was demonstrated for all sessions, even if the median score did not change.

**Table 3:** Median Self-Reported Confidence or Knowledge Scores Before and After Boot Camp Virtual Sessions Pre-/post-survey means were analyzed with the Wilcoxon rank-sum test (Mann-Whitney U test) for unpaired non-parametric data.

Session Title	Confidence or Knowledge Statement	Pre	Post	p-Value
Your Program Director	I can name several specific instances when I should go directly to the program director with an issue/concern.	3	4	<0.001
Chief Resident Expectations	I am knowledgeable of the role of a chief resident.	2	4	<0.001
After a Patient Dies	I know how to complete a death certificate	2	4	<0.001
Blood Products/Transfusion	I am confident that I know when to order certain blood products such as leukocyte reduced versus irradiated.	2	4	<0.001
Concussion/Head Trauma	I am comfortable with the protocol for on-field head or spinal trauma	2.5	4	<0.001
Common Clinical Scenarios	I am confident in managing common clinical scenarios.	3	4	<0.001
BEEP! A Night-on-Call: Nursing Pager Activity	I am confident in handling pages from a nurse while I am on call.	2.5	4	<0.001
Sign Out/Transitions of care	I am confident in signing out my patients to my peers.	3	4	<0.001
Admission Process and Writing Orders	I am confident in writing admission orders independently.	2	4	<0.001
Clinical Reasoning	I am confident in my ability to find and apply an appropriate illness script.	3	4	<0.001
Clinic/Ambulatory Tips	I am confident in caring for patients efficiently in the clinic setting.	3	4	<0.001
Clinical Documentation	I am confident in my clinical documentation.	3	4	<0.001
Teaching Students	I am confident in my teaching abilities.	4	4	=0.001
Telehealth	I am comfortable participating in telehealth as a health care provider.	3	4	<0.001

**Figure 2:**
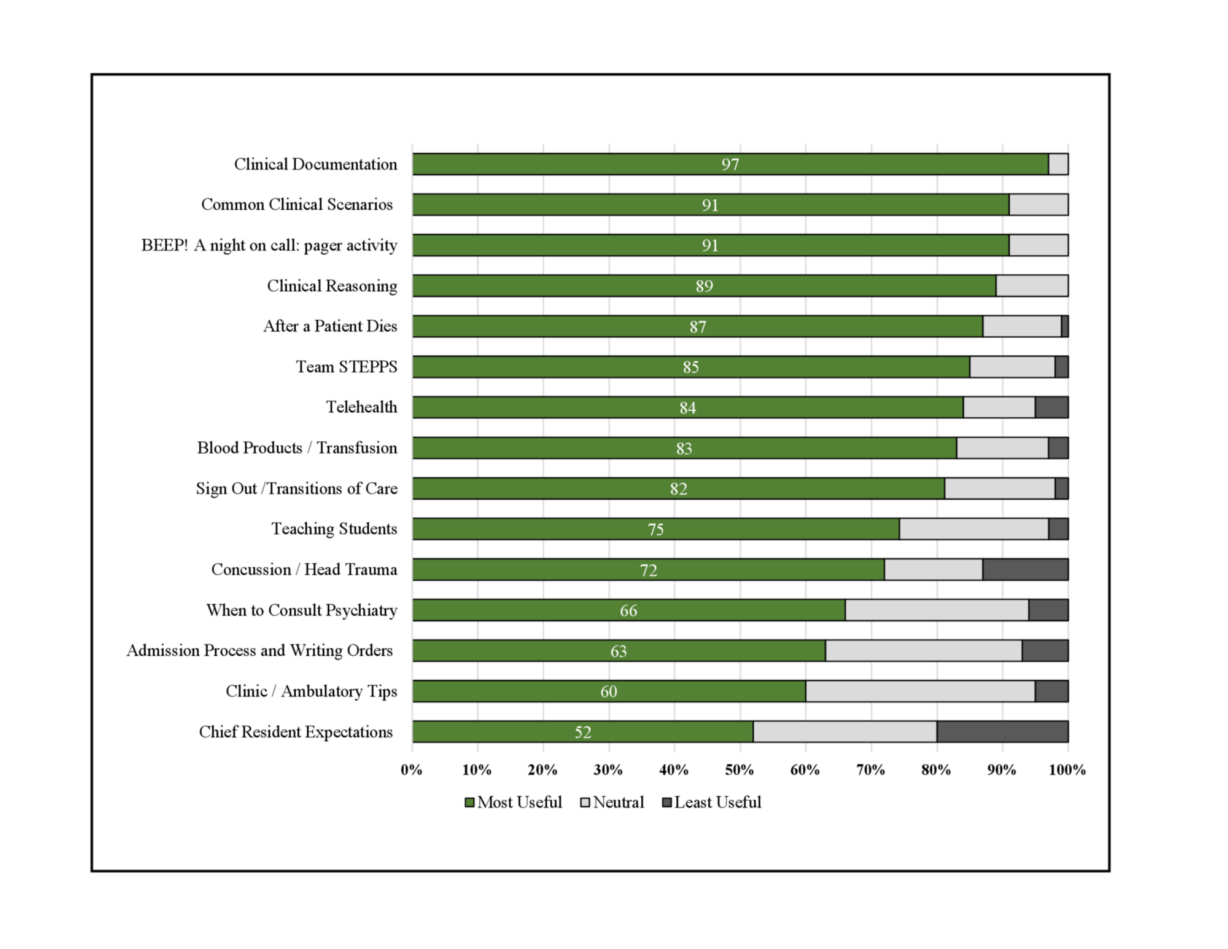
Student Evaluation of Virtual Teaching Sessions STEPPS, strategies and tools to enhance performance and patient safety

Ratings of session usefulness were collected for 15 of 26 sessions. Usefulness ratings are shown in Figure 2. Overall, all sessions analyzed were rated as useful or extremely useful by over half of the students (52-97%). The top five most useful sessions were the sessions on clinical documentation, common clinical scenarios, the night-on-call nursing paging activity, and clinical reasoning, which were rated as useful or extremely useful by 97%, 91%, 91%, and 89%, respectively. Only 4 of the 13 sessions received any feedback of “not useful at all”, and in all cases it was fewer than 3% of students.

The cumulative post-test was taken by 88 of 89 students. Of the students, 83 (94%) achieved a score of 70% or higher, 4 (4.5%) scored in the 60-70% range, and 1 scored 55%. Students were asked to provide feedback comments and state whether they believed certain sessions could remain virtual; comments are summarized in Table 5.

**Table 4:** Student Comments on the Virtual Non-Surgical Boot Camp Positive feedback was related to the flexibility of an online course and utility of the chat box, which, as some students reported, allowed them to be more engaged than they had been in live lectures. Constructive comments included the suggestion that sessions with a focus on patient care may be better achieved by a live session in coming years.

Positive comments
I enjoyed that this course was online. This is a big transition time for us, and it was very convenient to be able to have this online.
I am glad that this was online because it allowed me to go home and be with my family for the month.
The interactive nature of being able to type comments at any point and have your comments be addressed and interacted with by other students was helpful. I felt much more engaged than I normally do during in-person lectures.
Mandatory attendance and the chat box made it very interactive.
Virtual boot camp made it easier and less tiring to attend lecture since I could do it from home, and there was still great participation from the students.
Chat allowed other students to clarify things while the instructor was speaking, so the instructor didn't need to answer more questions.
Excellent resource that I plan on reviewing again the days prior to starting residency.
Constructive comments
I think it would have been helpful to have more clinically oriented courses in-person.
Anything that's super participation heavy should be done in person if possible, but lectures that are just lectures (with perhaps a poll here and there) are great to have online.
If you kept these lectures online, I would suggest shortening/separating them if possible. As a student, it is hard to pay full attention online with multiple hour-long lectures.
Summarizing handouts would be helpful to carry forward into residency.

Positive feedback was related to the flexibility of an online course and utility of the chat box, which, as some students reported, allowed them to be more engaged than they had been in live lectures. Constructive comments included the suggestion that sessions with a focus on patient care may be better achieved by a live session in coming years.

**Figure 3:**
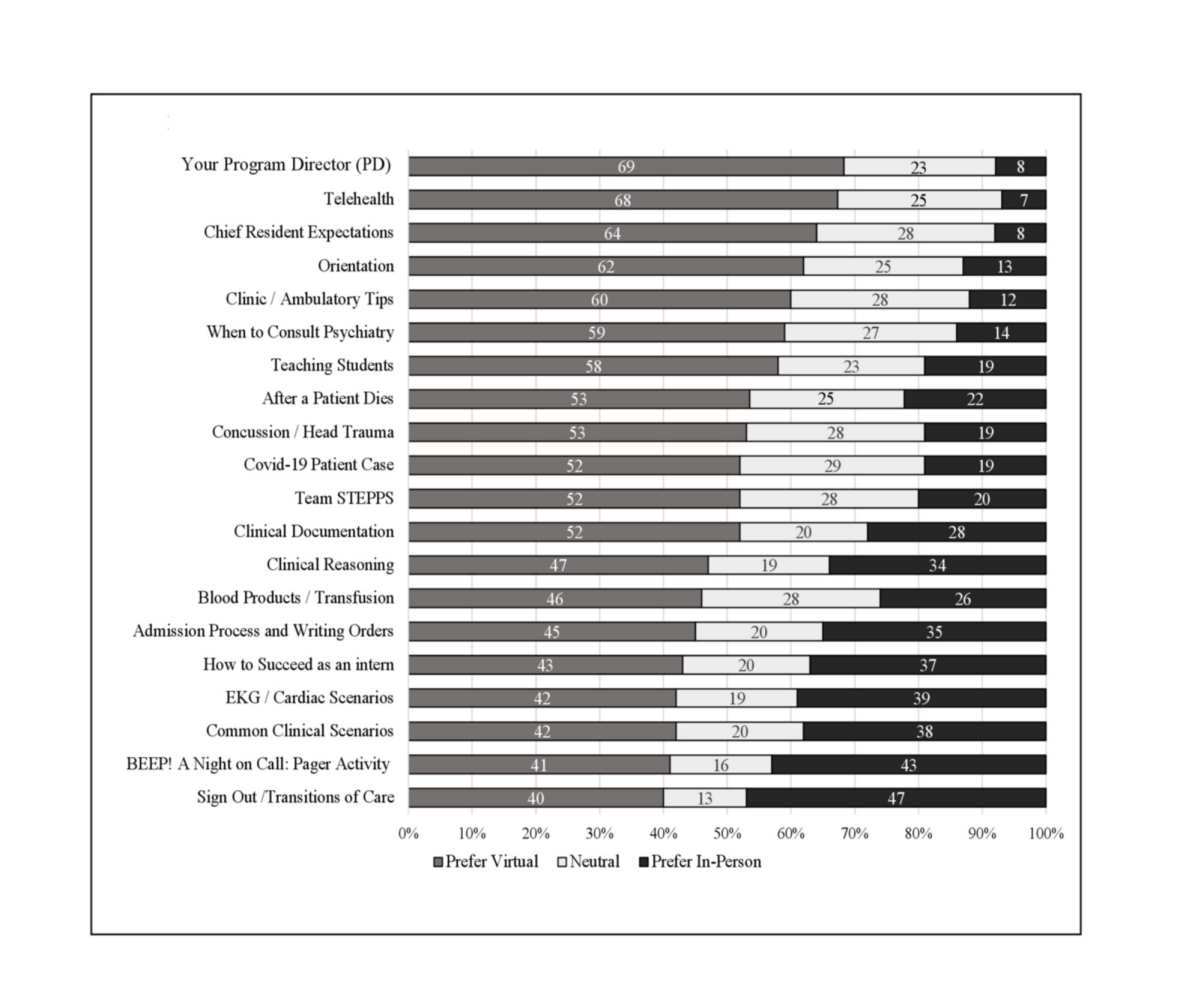
Student Preference for Boot Camp Format (Percent Preference for Virtual versus In Person) COVID-19, coronavirus disease 2019; STEPPS, strategies and tools to enhance performance and patient safety; EKG, electrocardiogram

Student preferences for which sessions could continue remotely versus which would be better facilitated in person are shown in Figure 3. For a majority of sessions, the percentage of students preferring a virtual format outnumbered those preferring an in-person session. The sessions most heavily focused on medical knowledge and patient care such as the clinical scenarios, sign-out activity, and night-on-call had the highest percentage of students preferring an in-person session.

## Discussion

This study demonstrates that students who participated in this elective non-surgical boot camp felt more confident and knowledgeable about handling situations they may encounter as interns and demonstrated an objective gain in knowledge beyond self-reported levels. Most of the teaching sessions were adapted to an online virtual synchronous format without difficulty, and feedback from students was overwhelmingly positive despite the change in format. Had there been more time to prepare, pagers could have been given to students to make the night-on-call activity more realistic; however, despite the absence of a pager, this was one of the highest-rated sessions. The course was well received with significant increases in student’s perceptions of readiness and comfort in multiple domains, which were linked to ACGME core competencies. Facilitators volunteered their time to construct and deliver the course content, and students used their personal computers; therefore, no high-fidelity simulation manikins or costly equipment was required. Although several studies on internship preparation courses have been published demonstrating increases in self-reported confidence, very few have included objective assessments such as the cumulative post-test included in our study. All students except for one achieved a score of above 60%, which would correlate with the score required to pass a national shelf examination.

Limitations of this remote activity include the fact it was a single-school study and that student’s level of interest and engagement may have been related to not having any other residency preparation options given the COVID-19 pandemic. The number of students who signed up for the course increased significantly in the seven days prior to the start of the course, which may reflect that. Other limitations include those of analyzing participation in pre- and post-surveys when some of the students did not complete both for each session. Lastly, it is a possibility that students who filled out their surveys were more engaged, and thus results were more likely to be positive.

## Conclusions

COVID-19 has presented many challenges to students and educators in undergraduate medicine. Now more than ever students need all the help they can and to feel more confident and prepared when they start internship on July 1. Despite the challenges we encountered, synchronous remote delivery of this boot camp was successful, and we will likely continue to hold certain sessions remotely in the future. This may be more useful for students traveling to arrange for new living arrangements during residency. Future work includes include scaling up to all 300 students and obtaining more volunteer facilitators that would enable small group breakout sessions which may increase opportunities to interact. We plan to re-survey students after their first month of internship to re-evaluate their perceived usefulness of this course. Overall, an online synchronous boot camp was rated as useful by students, helped them feel more confident about common topics encountered during residency, and demonstrated an appropriate gain in knowledge using a comprehensive post-course assessment. We were able to adapt our curriculum to a remote model and will likely keep several sessions in an online format in coming years based on positive feedback from students.
